# Determination of Functional Activity of Liver as Indicated by the Presence of Urobilinogen in the Urine

**Published:** 1913-04

**Authors:** 


Determination of Functional Activity of Liver as Indicated by the Presence of Urobilinogen in the Urine.—Allan Eustis, B. S., Ph. B., M. D., New Orleans *Medical and Surgical Journal,* December, 1912. While investigating several aniline dyes, Paul Ehrlich found that when an acid solution of paradimethylamidobenzylaldehyde was added to certain urines a bright red color was produced (*Munch Med. Woch.,* No. 15, 1901). Pappenheim (*Berliner Klinih Woch.,* No. 2, 1903), called attention to the occurrence of this reaction for urobilin, and Neubauer, the same year, demonstrated that the reaction is due to urobilingen, a colorless chromogen occurring in certain pathologic urines, attention to which was first called by Saillet (*Rev. de Med.,* No. 2, 1897), Richard Bauer (*Centralblat furinnere Med.,* No. 34, 1905) proved that the reaction is due to urobilingen, and as far as I can ascertain he was the first to call attention to its clinical significance. Bilirubin, as shown by Beck, Bauer and Fr. Muller, is reduced in the intestines to urobilingen, which in turn is reabsorbed by the portal circulation and transformed by the liver cells again into bilirubin, to be again excreted into the intestines as bilirubin in the bile. On exposure to air and light, urobilinogen is oxidized to urobilin, and the presence of urobilin is taken by some as an index of hepatic function.

The reaction is performed as follows: To ten cubic centimeters of freshly passed urine is added two or three drops of a solution of four Grs. of paradimethylamidobnzylaldehyde in 200 c. c. of 20 per cent. hydrochloric acid. In the presence of urobilinogen a scarlet red color is imparted to the urine, which persists on dilution with water. The red solution gives a definite absorption band in the spectrum in the orange-red between D.

and E., so that with the spectroscope one can detect very minute quantities of urobilinogen in the urine.

For clinical purposes, contrary to the opinion expressed by Sahli, I think the spectroscopic examination is not essential, as a little experience with urines in which it is strongly positive allows one to recognize the red color at once.

I have also observed that, while urines in which there is no urobilinogen may give a reddish color (due to pyrrol derivatives) on the addition of the reagent, on diluting with water the reddish tinge changes to yellow, while those urines giving a *positive* aldehyde reaction change to pink, the color persisting up to a very high dilution.

Beck demonstrated that urobilin is a normal constituent of dog's bile, but that if a biliary fistula is produced so that no bile can enter the intestines the urobilin in the bile gradually diminishes and finally disappears altogether, to reappear in the bile again when the animal is fed his own bile obtained from the fistula.

According to Bauer, if the common bile duct of a dog is ligated, in three hours the urine will give a positive aldehyde reaction; on the second day the urine will show the presence of bile pigments and still give the aldehyde reaction; on the third day there will be a very strong reaction for pigments, but only a slight aldehyde reaction, while on the fourth day there will still be a strong pigment reaction, but no aldehyde reaction. On feeding ox bile to the dog the aldehyde reaction will reappear.

What are the clinical points to be derived from these experiments? (1) The presence of the aldehyde reaction in the urine denotes lack of liver function, cirrhosis, carcinoma, etc., the degree of loss of function being denoted by the intensity of the reaction. (2) In a case of icterus from occlusion of the common duct, in which there has been an absence of the aldehyde reaction, if this suddenly reappears it denotes a reappearance of bile in the intestines or an overcoming of the obstruction. (3) In cases of diagnosed cholelithiasis in which for some reason operation is deferred, and in which the aldehyde reaction has been present, but has disappeared, and persists negative for several days, immediate operation is indicated to overcome the obstruction.

These facts have all been proved clinically by Muller, Bauer, Neubauer, Hildèrbrandt and others.

Below are tabulated the results of 363 cases in which the reaction was used, and in which a positive reaction was obtained in only those cases where there was evidence of interference with liver function:

| Disease. | No. Cases Observed. | Aldehyde Reaction. | |
|---|---|---|---|
| | | No. Pos. | No. Neg. |
| Abscess of liver (amebic) | 2 | 1 | 1 |
| Acute bronchitis | 10 | 0 | 10 |
| Acute catarrhal jaundice | 4 | 2 | 2 |
| Acute diarrhea | 15 | 2 | 13 |
| Acute parotitis | 15 | 0 | 15 |
| Alimentary glycosuria | 5 | 2 | 3 |
| Aneurism (thoracic) | 4 | 2 | 2 |
| Amebic dysentery | 2 | 0 | 2 |
| Appendicitis (acute) | 3 | 0 | 3 |
| Appendicitis (chronic) | 2 | 0 | 2 |
| Arteriosclerosis | 39 | 25 | 14 |
| Arthritis deformans | 4 | 1 | 3 |
| Banti's disease | 2 | 0 | 2 |
| Cholethiasis (common duct) | 2 | 2 | 0 |
| Cholethiasis (simple cystic) | 2 | 0 | 2 |
| Chronic gonorrhea | 5 | 0 | 5 |
| Chronic intestinal toxemia | 45 | 10 | 35 |
| Chronic passive congestion of liver (cardiac disease) | 4 | 4 | 0 |
| Chronic valvular heart disease | 59 | 4 | 55 |
| Cirrhosis of liver (alcoholic) | 6 | 6 | 0 |
| Cystitis | 5 | 0 | 5 |
| Diabetes mellitus | 8 | 2 | 6 |
| Eczema | 2 | 0 | 2 |
| Erythema multiforme | 2 | 0 | 2 |
| Exophthalmic goitre | 3 | 0 | 3 |
| Malaria fever, tertian | 6 | 2 | 4 |
| Myxedema | 1 | 0 | 1 |
| Nephritis, chronic parenchymatous | 3 | 0 | 3 |
| Nephritis, chronic interstitial | 10 | 5 | 5 |
| Peritonitis, tubercular | 2 | 0 | 2 |
| Pernicious anemia | 1 | 0 | 1 |
| Pneumonia, lobar | 2 | 0 | 2 |
| Pneumonia, lobuar | 3 | 0 | 3 |
| Rheumatism, acute articular | 2 | 0 | 2 |
| Rheumatoid arthritis | 9 | 3 | 6 |
| Scarlatina | 2 | 0 | 2 |
| Syphilis, primary | 3 | 0 | 3 |
| Syphilis, secondary | 5 | 2 | 3 |
| Syphilis, tertiary | 15 | 6 | 9 |
| Tinea circinata | 3 | 0 | 3 |
| Tinea saginata | 2 | 0 | 2 |

| | | | |
|---|---|---|---|
| Typhoid fever.................. | 4 | 0 | 4 |
| Tonsilitis, acute follicular........ | 4 | 0 | 4 |
| Tuberculosis, pulmonary......... | 20 | 2 | 18 |
| Ulcer of stomach............... | 3 | 0 | 3 |
| Uncinariasis .................... | 8 | 2 | 6 |
| Urticaria . .................... | 3 | 2 | 1 |
| Vincent's angina............... | 2 | 0 | 2 |
| | 363 | 87 | 276 |

